# Determining the influence of livelihoods, land access, and location on household food and nutrition security in KwaZulu-Natal, South Africa

**DOI:** 10.1186/s40100-025-00435-w

**Published:** 2025-11-15

**Authors:** Mallika Sardeshpande, Rowan Naicker, Sithabile Hlahla, Onisimo Mutanga, Rob Slotow, Tafadzwanashe Mabhaudhi

**Affiliations:** 1 Centre for Transformative Agriculture and Food Systems, University of KwaZulu-Natal, Pietermaritzburg, South Africa; 2 School of Agriculture and Science, Discipline of Geography, University of KwaZulu-Natal, Pietermaritzburg, South Africa; 3 Future Water Institute, University of Cape Town, Cape Town, South Africa; 4 Oppenheimer Fellow in Functional Biodiversity, Centre for Functional Biodiversity, School of Life Sciences, University of KwaZulu-Natal, Pietermaritzburg, South Africa; 5 Centre on Climate Change and Planetary Health, London School of Hygiene and Tropical Medicine, London, United Kingdom

**Keywords:** Agriculture, Dietary diversity, Food sovereignty, Greenspace, Nutrition transition, Rural–Urban gradient

## Abstract

Access to healthy food varies across socioeconomic circumstances such as primary livelihood, and access to shops, land, farms, and cities. We compared household food and nutrition security across households with food-producing and non-food-producing livelihoods, access to farms and non-farm greenspaces, and their location relative to farms, non-farm greenspaces, shops, and urban centres. We used household survey data and landscape spatial data from five sites across the socioeconomic gradient. Food security was higher in wealthier, more educated, urban households, and dietary diversity was highest in rural and agrarian households. Non-farm greenspaces such as communal and informal land, and backyard and communal gardens, were significant food sources. On-farm diversification, smallholder support, and provisioning of non-farm greenspaces can improve food and nutrition security. Agricultural extension and spatial planning policy should target smallholder capacitation, and development of fruit and vegetable gardens in both rural and urban areas, for sustainable, equitable, healthy food environments.

**Graphic abstract:**

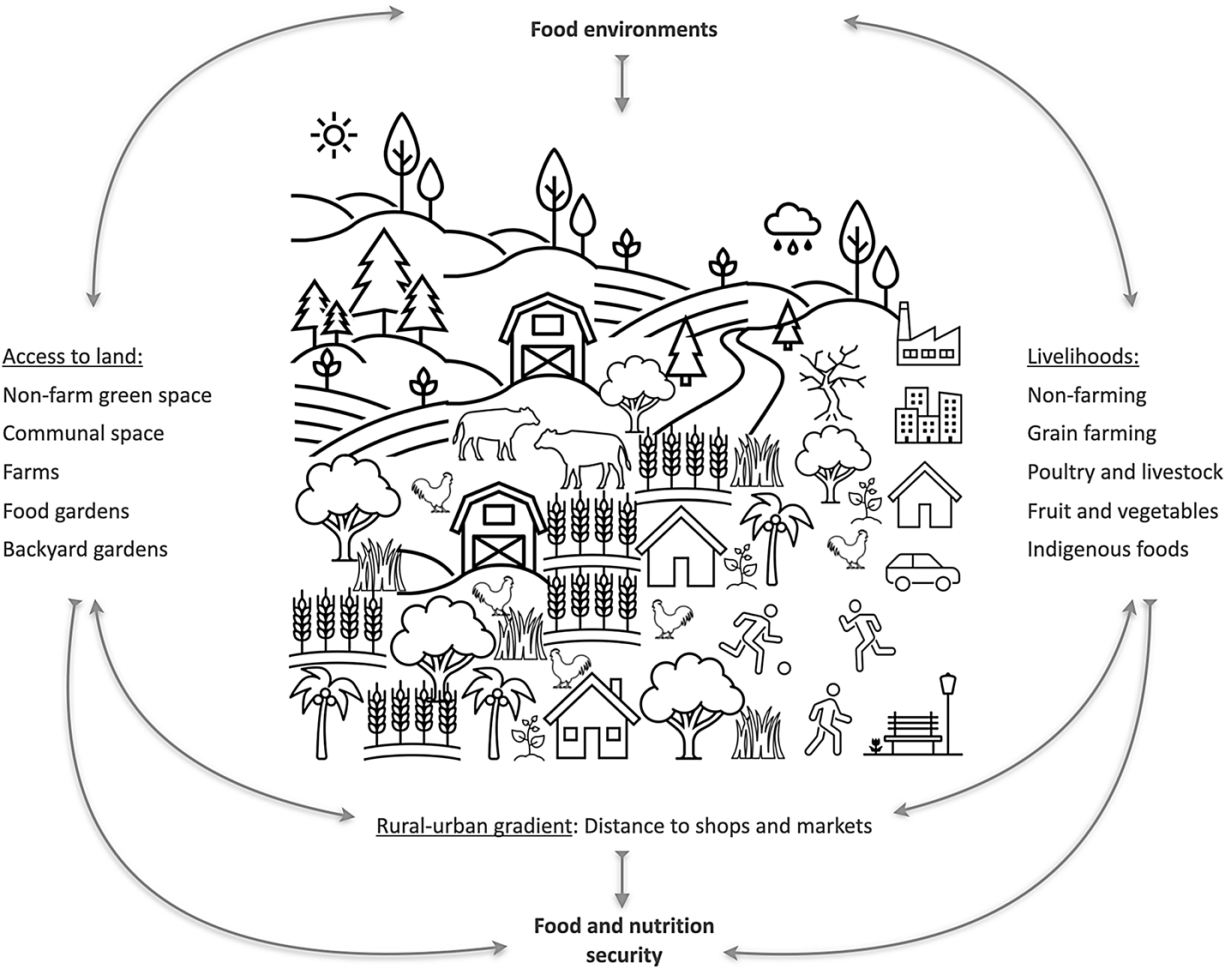

## Introduction

The rise of industrial agriculture and global trade has led to significant changes in food production and consumption; however, the increase in global food stocks has not addressed local inequalities in food availability, dietary choices, and nutrition security (Brouwer et al. 2020). Streamlining for efficiency has resulted in food production, supply chains, and consumer diets becoming uniform and limited in nutrition and quality (Pereira & Drimie 2016). On the production end, farmers often transition to resource-intensive monocultures at the expense of local traditional crops, to sustain their livelihoods in highly competitive land and food markets (Mustafa et al. 2021). At the consumer level, greater availability and affordability of calorie-dense, nutrient-poor foods than fresh local foods can influence dietary choices, and erode nutrition security (Turner et al. 2018). Food and nutrition security comprise seven components which promote an active and healthy life. These include access, availability, utilisation, and stability of food resources, which should be diverse, of high quality and safe for consumption (Marivoet et al. 2019). These components often vary across socioeconomic contexts, such as economic activities (livelihoods), access to capital and land tenure, location with respect to roads, etc.

Food-producing livelihoods vary in scale; while only one percent of all farms occupy 70% of the world’s food-producing land, more than three-quarters of all farms worldwide consist of smallholdings (Lowder et al. 2021). Even though large-scale agriculture produces over half of the world’s food supply, it utilises a limited range of crop and animal species. Smallholder farms, which contribute about a third of the world’s food supply on a quarter of the world’s farmland, also host greater crop diversity and low-waste food economies (Ricciardi et al. 2018). However, for most smallholder farmers, food-producing livelihoods do not necessarily contribute to their household’s food, nutrition, and income security. For example, large-scale farming may debilitate smallholder farmers’ sovereignty by capturing land and food markets, and through downstream ecological impacts of industrial farming (Ahmed et al. 2019, Rockstrom et al. 2020). In cases where smallholder farming cannot contribute to food and income sufficiency, agrarian households become less capable of either procuring fresh and nutritious foods from their own farms, or affording them at the local markets (Popkin et al. 2012, Development Initiatives 2020). This, in turn, can exacerbate and perpetuate nutrition and health inequalities (Rohr et al. 2019; Tomita et al. 2020).

Access to land takes various forms and can significantly affect households’ and communities’ food and nutrition security outcomes. For example, securing land tenure can help food producers improve land productivity and devise strategies to adapt to environmental change (Tseng et al. 2021; Murken and Gornott 2022). Dynamic communal arrangements can foster multifunctional landscapes that produce diverse seasonal, locally adapted foods, and useful products (e.g. fibre, fodder, medicine) year-round (Genin et al. 2018). Besides designated farmland, access to formal and informal greenspaces can be a significant food source for households. Formal greenspaces, like allotments and communal food gardens, can enhance fresh produce availability within the local food economy (Kanosvamhira and Tevera 2022; Suchá and Dušková 2022). Such spaces are especially important when market penetration is poor, or the terrain does not permit intensive food production. Informal and domestic greenspaces from which food is procured include vacant lots and home gardens, where food is gathered or grown to benefit local households (Sardeshpande In Press). By supplementing food supply and dietary diversity, these greenspaces can contribute significantly to low-income areas, or urban and peri-urban areas where land use is highly contested (Chakona and Shackleton 2019). In this article, the word greenspaces denotes non-farm food-producing land, and informal land, including publicly accessible fallows.

The feasibility of food-producing livelihoods and access to land changes along geographic gradients, most notably along the rural–urban continuum (Gebre and Gebremedhin 2019; Giller et al. 2021). In addition, the food procurement pathways also change along this gradient, with urban populations more reliant on food purchases from shops than rural agrarian counterparts. Urban populations, especially the urban poor, are vulnerable to food and nutrition insecurity due to their lifestyles, lack of access to land and/or affordable food, and subsequent dependence on externally imported, processed, and prepared foods (Haggblade et al. 2017; Haysom and Tawodzera 2018). The de-linking of livelihoods from food production and greenspaces can compromise knowledge about dietary diversity and nutrition quality, driving preferences instead towards convenience, satiety, marketing, and food affordability (Webb et al. 2023). Emerging research calls for decentralised and diverse food systems, which can be implemented at various scales, from personal and community food environments to local food economies and higher-level food systems governance (El Bilali et al. 2019; Fanzo et al. 2020).

In this context, our study aims to explore the relationships between household food and nutritional security and different types of livelihoods, access to different types of land, and location with respect to different sources of food, across the rural–urban gradient in the province of KwaZulu-Natal, South Africa. South Africa continues to experience increasing household food insecurity, despite the country being food secure at a national level (Bjornlund et al. 2022). The results from this study can inform ongoing policy efforts to strengthen local food systems and household food and nutrition security, allowing for a paradigm shift from a focus on a national food system and food security. Previous research on food environments has investigated specific aspects such as the influence of food retailers on dietary choices (Rombach et al. 2023) and diet-related disease (Otterbach et al. 2021); of farm-related livelihoods and on-farm diversity with household nutrition outcomes (Ricciardi et al. 2018; Ickowitz et al. 2019); or of wild food accessibility on dietary diversity (Cooper et al. 2018; Chakona and Shackleton 2019). This investigation is unique in that it combines spatial data with socioeconomic data, spanning a range of food environments (rural–urban, built-wild, e.g. Downs et al. 2020). Further, this study develops a food proximity index calculated based on respondent-reported frequency of procurement of food from sources that are close (e.g.backyard, local farm) and distal (e.g. supermarket, large chain store). This is a novel approach that aims to test to what extent household food procurement strategies are linked to availability and accessibility of different types of food sources. Understanding how food and nutrition security relates to livelihoods associated with food production, or not, access to different types of non-farm non-built land, and household location along the rural–urban gradient, can help formulate appropriate policy and practice responses to specific contexts at scale.

### Research questions

In this paper, we explore the relationships between food and nutrition security and types of food-producing livelihoods (livestock, grain, fruit and vegetables), access to food-producing land (private, communal, public), and location with respect to food sources (shops, non-farm greenspaces, farms), and along the rural–urban gradient. We use the Household Food Insecurity and Access Scale (HFIAS) (Coates et al. 2007) to measure food security, the Household Dietary Diversity Score (HDDS) as a proxy for nutrient adequacy of the diet of individuals (Kennedy et al. 2013) (nutrition security), and GIS to measure distances between households and identified food sources. The research questions were:

(i) Does involvement in certain types of food-producing livelihoods predict household food security, dietary diversity, or location?
(ii) Does access to certain types of food-producing land predict household food security, dietary diversity, or location?
(iii) Does household location with respect to food sources, and along the rural–urban continuum, predict food security or dietary diversity?

This understanding will be critical in developing policies and practical, real-world interventions that key stakeholders can implement to improve food and nutrition security in South Africa.

### The South African context

South Africa faces multiple challenges in the quest for food and nutrition security, including poverty and inequality, water scarcity, soil degradation, climate change, civil unrest, epidemic outbreaks, and the legacy of apartheid (van der Berg et al. 2022). About 33% of the population is unemployed, and about 47% of the population receives social grants from the government for child care, disability, unemployment, and social distress (Patel et al. 2023). Historically, apartheid policies forcibly placed indigenous communities in regions usually characterized by marginal farming lands, including infertile soils and limited water supplies. This geographical segregation severely restricted these communities’ options for engaging in agricultural activities, leaving them with few choices regarding farming. Further compounding this problem was the lack of market development and economic opportunities, limiting consumer access to fresh food (Tshishonga 2020).

Consequently, the inability to produce or purchase nutritious foods has contributed to the triple burden of undernutrition, malnutrition, and diet-related diseases, and has had profound long-term effects on people’s health and well-being (Masters et al. 2022). A comprehensive, multifaceted approach is needed to address the complex food and nutrition security issues in South Africa. A fundamental component within this process is to address the structural inequalities affecting previously marginalized communities. This includes improving access to sources of fresh food and investing in infrastructure to connect these areas to food markets and other economic opportunities (Boatemaa et al. 2018).

Moreover, adopting environmentally and socially adaptive agrarian systems is necessary to ensure people not only survive, but thrive in a rapidly changing climate (Tambe et al. 2023). Thus, promoting food sovereignty and promoting locally grown and adapted food can help improve people’s access to a variety of nutritious foods, which is essential for achieving food and nutrition security. Studies (Hobbs 2020; Hendry et al. 2019) have demonstrated that locally-produced food, with smaller supply chains, can provide communities with the ability to control and influence the food that they consume. Consequently, it is essential to explore the relationships between food and nutrition security, the types of food-producing livelihoods, and communities’ relative access to food-producing land. By addressing these concerns, we hope to better understand the complex interplay between food and nutrition security, food-producing livelihoods and constraints on these, and access to land and food sources.

## Methods

### Data collection

**Fig. 1:**
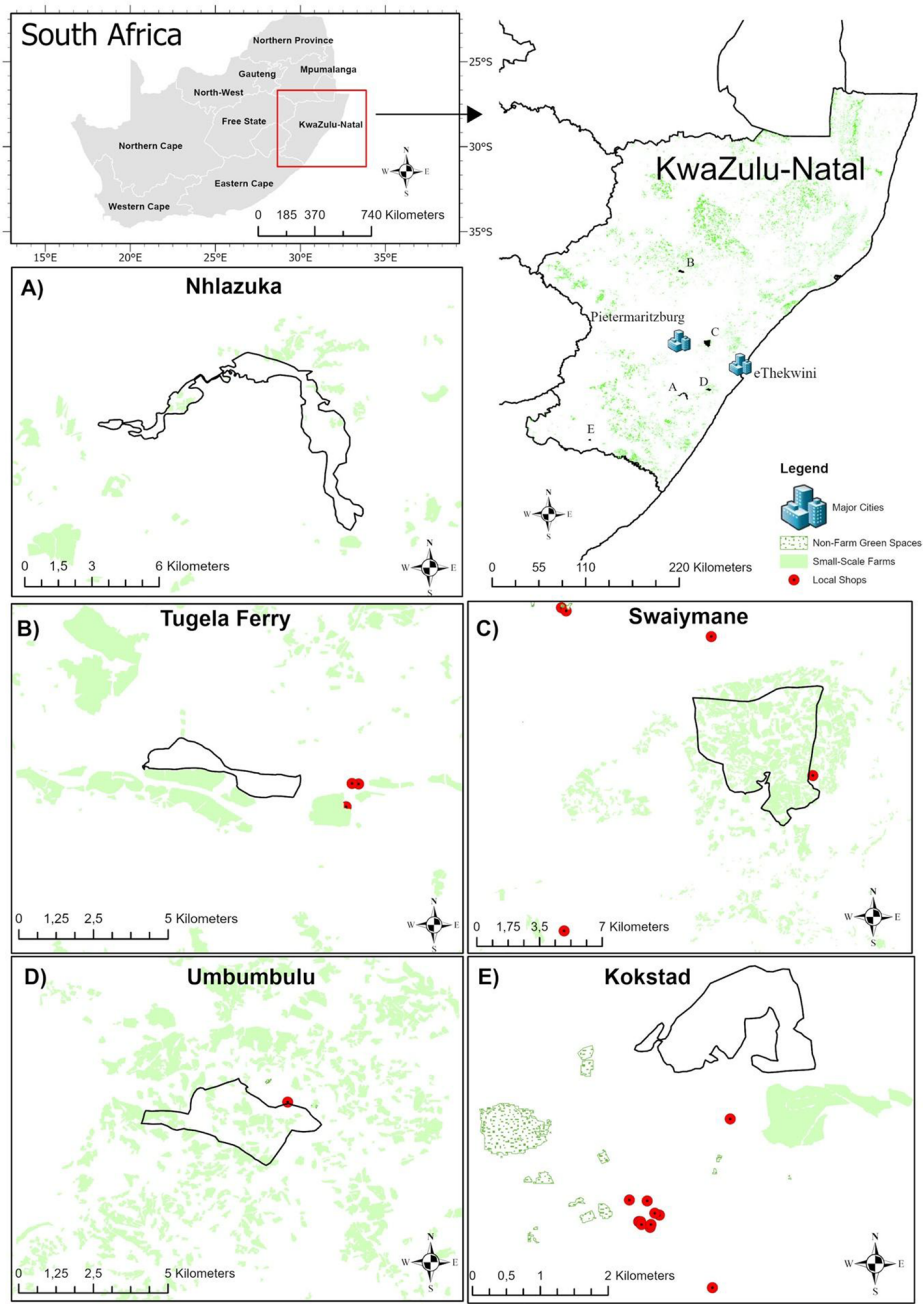
Spatial distribution of the different study sites in KwaZulu-Natal, South Africa, in relation to non-farm green spaces, small-scale farms, and local shops. Where certain features are not visible (e.g. major cities, non-farm green spaces, local shops), they are located beyond the spatial scale of the panel

Data were collected from October–November 2019 using a questionnaire survey at five study sites in KwaZulu-Natal, South Africa (Fig. 1 and Table 1). These sites were part of extensive work carried out by the research team under the Sustainable and Healthy Food Systems project (Hawkins et al. 2022; Khowa et al. 2023). The data collected during these studies included demographic information; water use and sanitation; food preparation and hygiene; food and nutrition security; land, livestock and crops; chicken ownership and consumption; food choices, food safety; indigenous knowledge and ecosystem services. The study was ethically reviewed and approved by the Humanities and Social Sciences Research Ethics Committee of the University of KwaZulu-Natal (Ref. No. HSS0287/018).

**Table 1:** Description of study sample sites

Site (location)	Population	Households	Surveyed households	Main local livelihoods
Nhlazuka – Fig. 1A (Rural, 90 km from urban centre)	15,104	1774	97	Subsistence farming (maize, sorghum, groundnut, taro, cabbage), brick-making, forest-based livelihoods
Tugela Ferry – Fig. 1B (Rural, 120 km from urban centre)	2093	440	100	Animal husbandry and crop production
Swayimane – Fig. 1C (Rural, 40 km from urban centre)	6857	1372	99	Crop production (maize, beans, taro, sweet potato, sugarcane) and animal husbandry
Umbumbulu – Fig. 1D (Peri-urban, 40 km from city centre)	2684	416	92	Crop production and animal husbandry
Kokstad – Fig. 1E (Urban, Town)	51,561	8867	98	Dairy farming and processing, agriculture

#### Questionnaire survey

Fifty field assistants who had completed secondary education, and were fluent in the local language, isiZulu, were hired and trained to administer a semi-structured questionnaire. The assistants lived within the communities and ten were selected from each community. A convenience sampling technique was adopted, as the households were selected based on their availability and willingness to participate in the survey. In an attempt to ensure that the questionnaire was distributed as widely as possible to the different areas in each community, the field assistants were encouraged to work in different parts of the communities, as far apart as possible. The assistants went door-to-door and only interviewed willing participants. Where a participant refused to participate, the field assistant proceeded to the next house. The final sample consisted of 486 households, and an individual from each household was interviewed. Each interview was conducted with the self-identified head of the household. Where the head of the household was unavailable, the interview was conducted with whoever was available, but above 16 years of age. Before responding to any questions, the respondents were informed of the study and provided verbal informed consent to their participation.

#### Mapping

Representative GPS coordinates of each respondent household and associated attributes were recorded in an Excel document and imported into an ArcGIS environment. A 2021 land cover map was obtained from the South African D.F.F.E (South African Department of Forestry, Fisheries and Environment). The landcover map (with an overall model accuracy of 80%) was developed over a multi-temporal period using 20 m Sentinel-2 satellite imagery (South African Department of Forestry, Fisheries and Environment 2021). The distribution of small-scale farms and non-farm greenspaces (comprising recreational fields and other areas) was then extracted from the landcover map. After that, the precise location of every shop was derived through an exhaustive geo-tagging procedure (e.g. Otterbach et al. 2021). Our definition of shops encompassed built food environments including street vendors, kiosks, retailers, farmers markets, supermarkets, and hypermarkets (following Downs et al. 2020). Each shop within a 20 km radius from the centre of each study site polygon was tagged using the Google Earth Pro platform. Euclidean distances of each household to greenspaces, farms and shops were derived using the “Spatial analyst tool” in ArcGIS 10.7. This distribution was then used to explore the relationship between food security and dietary diversity.

### Data analyses

**Fig. 2:**
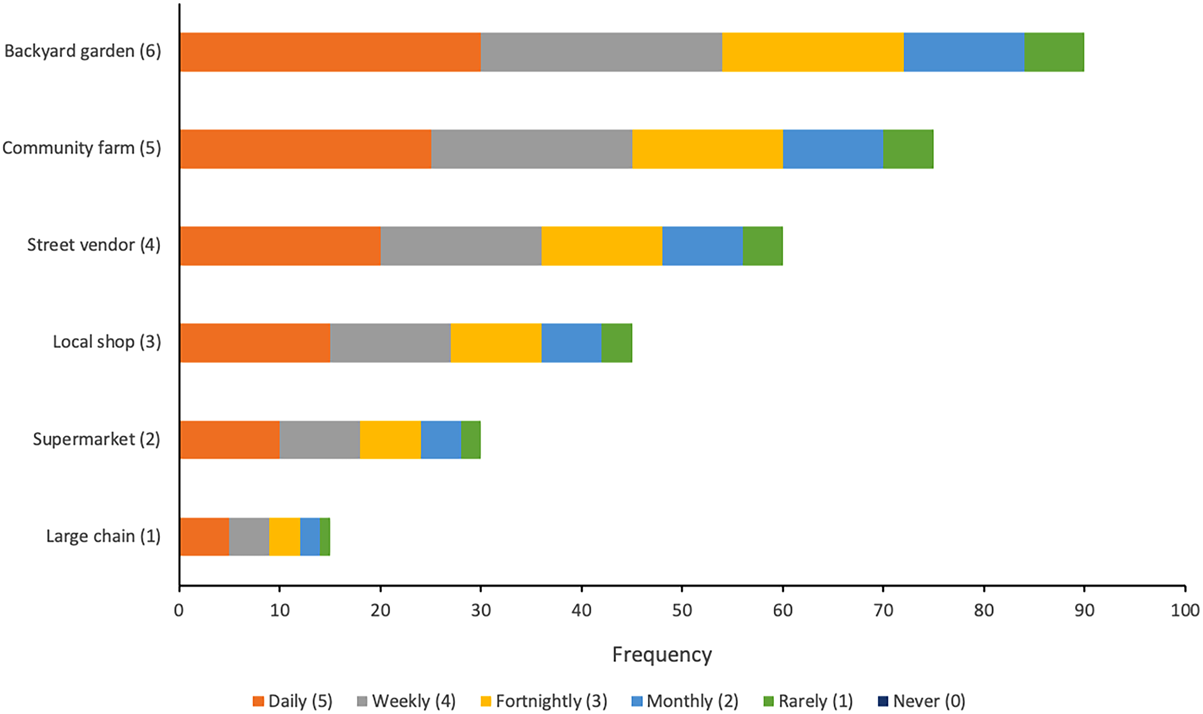
Weighted sources and frequencies for food procurement used to calculate the food proximity index

Data variables relevant to household food and nutrition security, livelihoods, and land use were extracted from the dataset for analysis (Appendix Table 6). Household size was calculated by adding the numbers of adult and child male and female members. Next, household income was classified based on the data collection categories, i.e., the distribution of the sample. Households earning ≤ ZAR 5000 annually were classed in the first quintile, and those earning > ZAR 20,000 annually were in the fifth quintile. Respondent education was assigned ranks, with no education being 0, and tertiary education being 3. Household food security and nutrition security were calculated as per the Household Food Insecurity and Access Scale (HFIAS) framework (Coates et al. 2007), and the Household Dietary Diversity Score (HDDS) framework (Kennedy et al. 2013) of the Food and Agricultural Organisation, respectively. Both these scores are incremental, such that higher scores imply greater food and nutrition security. A food proximity index was calculated by combining the frequency of food procurement from different sources, as reported in the questionnaire (Fig. 2). Respondents in the survey named up to six food sources, including backyards, communal farms, local informal vendors, local shops, supermarkets, and large chain stores. We assumed that proximity to food sources is at its maximum in the backyard garden and minimum in a large chain store that may import food nationally and globally. Frequency of procurement was assigned linear ranks, with daily procurement assigned maximum weight. Frequency and proximity scores were multiplied per category per household, and added to yield the food proximity index. The food proximity index is derived from respondent information on household food consumption strategies, and represents an important independent aspect of household location that spatial-based assessments may not capture.

Due to the non-normal distribution of most variables, non-parametric tests were used to analyse the data (Appendix Table 6) in R 4.0.4 (R Core Team 2021). Kruskal–Wallis Tests were used to compare medians of household food security, dietary diversity, and food proximity scores across the different survey sites, household income quintiles, and respondent education level, as well as frequencies of food procurement across different sources. The null hypothesis for these tests was that there was no significant difference in household food security, dietary diversity, and food proximity scores across survey sites, household income quintiles, and respondent educational attainment. Spearman-rank correlation tests were run to test for collinearity between continuous household variables, namely food security, dietary diversity, and food proximity scores, and distances to farms, greenspaces, and shops. Because correlations between variables were weak, all variables were used in regression models with no detriment to the predictive power or estimates of the models (Kutner et al. 2005). Binomial logistic regression models were fitted to compare food security, dietary diversity, and food proximity scores between households that did and did not undertake (any) food-producing livelihoods (research question i), and between households that had and did not have access to different types of food-producing land (research question ii). The models investigated the influence of livelihoods (both agricultural and non-agricultural), and access to (four) different types of land, on household food security, dietary diversity, and food proximity scores. Pearson’s Chi-squared tests were used to detect dependencies between household food-producing livelihoods and land access. The null hypothesis for these tests was that household food production activities were independent of types of land accessed. Poisson regression models were fitted to determine household and location predictors of food security and dietary diversity (research question iii). These models investigated the influence of household size, wealth, location, and respondent age and education, on household food security, dietary diversity, and food proximity scores.

## Results

### Socio-economic data

A total of 486 complete responses were collected from five sites, with a mean of 97 households per site (± 3). The average age of the respondent was 45 years (± 14), and the average household size was 7 members (± 5). Over half of the households surveyed were headed by female members, and over a fifth by male members, with the balance headed jointly by both (Appendix Table 7). Over a third of the households reported to be government grant recipients, and over a quarter relied on these grants for their primary income. About a fifth of the surveyed households each cited their primary source of income as full or part-time employment, and pension. Some 60% of the surveyed households engaged in food-producing livelihoods. The average distance from households to food provisioning shops was 7.6 km (± 10), to greenspaces was 12.8 km (± 10), and to farms was 0.5 km (± 0.5).

### Household food security, dietary diversity, and food proximity scores

Household food security, dietary diversity, and food proximity scores varied significantly across sites (Table 2). Whereas Kokstad had the highest mean food security score, Swayimane had the highest mean dietary diversity score and food proximity index. Across respondent education levels, scores varied significantly for household food security and food proximity, but not dietary diversity. Respondents with tertiary education had higher food security scores, and those with primary education had higher food proximity scores. Household food security scores also varied significantly for household wealth quintiles, with the highest scores among the wealthiest quintile. There were weak significant correlations between household food security, dietary diversity, and food proximity scores, and distances to farms, greenspaces, and shops (Appendix Table 20).

**Table 2:** Means, standard deviations, and Kruskal–Wallis Test statistics of household food security scores, dietary diversity scores, and food proximity indices by site, education level, and wealth quintile (*N* = 486)

Variable	Category	Food security score		Dietary diversity score		Food proximity index	
		Mean	sd	Mean	sd	Mean	sd
Sites (nominal)	Kokstad	22.29	4.97	9.23	2.54	36.24	21.83
	Nhalzuka	20.52	5.88	8.46	3.47	29.5	20.03
	Swayimane	19.96	5.10	10.63	2.04	46.58	22.58
	Tugela Ferry	19.95	5.43	9.33	3.76	31.51	15.42
	Umbumbulu	21.64	4.23	9.36	4.16	31.65	19.58
Kruskal–Wallis AnoVa across sites		χ^2^ = 17.58, df = 4, *p* = 0.002		χ^2^ = 38.1, df = 4, *p* < 0.001		χ^2^ = 39.99, df = 4, * p* < 0.001	
Education level (ordinal)	0	19.76	5.98	9.48	3.53	29.87	19.47
	1	20.57	5.10	9.33	3.39	36.85	20.54
	2	20.94	4.95	9.19	3.46	36.15	21.11
	3	22.15	5.28	10.04	2.59	34.4	21.82
Kruskal–Wallis AnoVa across education level		χ^2^ = 8.97, df = 3, * p* = 0.03		Insignificant		χ^2^ = 7.77, df = 3, * p* = 0.05	
Wealth quintile (ordinal)	1	17.86	7.48	9.73	2.69	28.27	15.93
	2	18.12	6.15	9.82	2.30	37.46	22.72
	3	20.74	6.13	9.59	3.33	38.29	25.64
	4	20.9	4.67	8.84	4.03	33.73	20.13
	5	22.19	4.05	9.62	3.04	35.23	19.36
Kruskal–Wallis AnoVa across wealth quintile		χ^2^ = 27.61, df = 4, * p* < 0.001		Insignificant		Insignificant	

### Livelihoods: non-agrarian, food-producing, and sub-types

Binomial logistic regression models are presented in Appendix Tables 8–12, and significant results from each model are presented in Table 3. Respondent age had small incremental effects on livelihoods, with older respondents more likely to be in households engaging in agricultural production in general (2%), and production of grains (5%) and poultry (3%) in particular. Respondent education had a significant incremental effect on households producing grains (60%), and a detrimental effect on those producing fruit and vegetables (34%). Households with more members were slightly less likely to produce fruits and vegetables (7%), and households with greater dietary diversity were less likely to produce poultry (12%). For every kilometre increase in distance to shops, there was a slight increase in the likelihood of households engaging in livestock (4%), grain (5%), and poultry (6%) production. Similarly, distance to greenspaces had an incremental effect on household engagement in agriculture (6%), particularly grain production (6%). Households situated away from farms were significantly less likely to engage in the production of grains (37%) and poultry (83%).

**Table 3:** Significant household predictors of different types of food-producing livelihoods. The 1-pχ^2^ value indicates Chi-squared goodness of fit for the model, and only significant predictors (*p* < 0.05) are reported for each model in this table. Full statistics of each regression model are presented in Appendix Tables 8–12

Livelihood (independent variable)	1-pχ^2^	Household attribute (predictor variable)	z	p-value	Log (estimate)
Agriculture (any sub-type)	6.92E-06	Respondent age	2.168	0.05	1.018
		Distance to greenspaces	4.514	0.001	1.061
Livestock production	0.01719307	Distance to shops	2.693	0.01	1.043
Poultry production	1.05E-05	Respondent age	2.187	0.05	1.026
		Dietary diversity	− 3.270	0.01	1.123
		Distance to shops	3.630	0.001	1.060
		Distance to farms	− 2.010	0.05	1.829
Grain production	2.34E-14	Respondent age	4.949	0.001	1.048
		Respondent education	3.408	0.001	1.600
		Distance to shops	− 3.341	0.001	1.047
		Distance to greenspaces	4.083	0.001	1.056
		Distance to farms	− 3.997	0.001	2.777
Fruit and vegetable production	0.02702448	Household size	− 2.232	0.05	1.070
		Respondent education	− 2.068	0.05	1.344

### Land access: private, communal, public

Pearson’s Chi-squared tests were conducted to discern whether food-producing livelihood activities were independent of greenspaces used to procure food. The results (Table 4) highlight that users of backyard gardens, communal gardens, and communal lands tend to produce livestock, poultry, and fruits and vegetables at frequencies greater than expected of independent samples. Users of informal lands also tend to engage in food production overall more than would be expected of independent samples. Due to small observed frequencies, tests weren’t run for users of verges (*n* = 6) and school gardens (*n* = 5). Some of these independences were further quantified in the binomial logistic regression models.

**Table 4:** Pearson’s Chi-squared statistics and significance for test of independence between food production activities and greenspace use

Livelihood	Land use	χ^2^	p-value	df
Agriculture (any sub-type)	Informal Lands	0.0024772	0.9603	1
Livestock farming	Informal Lands	0.71468	0.3979	1
Poultry farming	Informal Lands	1.58E-28	1	1
Grain farming	Informal Lands	3.3966	0.06533	1
Fruit and Vegetable farming	Informal Lands	0.23606	0.6271	1
Agriculture (any sub-type)	Communal Lands	12.747	**0.0003565**	1
Livestock farming	Communal Lands	9.0952	**0.002563**	1
Poultry farming	Communal Lands	5.1371	**0.02342**	1
Grain farming	Communal Lands	4.0106	**0.04522**	1
Fruit and Vegetable farming	Communal Lands	0.06647	0.7965	1
Agriculture (any sub-type)	Community Gardens	22.734	**1.86E-06**	1
Livestock farming	Community Gardens	4.6947	**0.03026**	1
Poultry farming	Community Gardens	11.948	**0.0005471**	1
Grain farming	Community Gardens	8.7466	**0.003102**	1
Fruit and Vegetable farming	Community Gardens	26.104	**3.24E-07**	1
Agriculture (any sub-type)	Backyard Gardens	61.096	**5.44E-15**	1
Livestock farming	Backyard Gardens	6.7844	**0.009196**	1
Poultry farming	Backyard Gardens	24.095	**9.17E-07**	1
Grain farming	Backyard Gardens	13.724	**2.12E-04**	1
Fruit and Vegetable farming	Backyard Gardens	34.587	**4.08E-09**	1

The bold values indicate a p-value of < 0.05, which renders the relationship statistically significant

Binomial logistic regression models to determine land access predictors are presented in Appendix Tables 13–17, and significant results from each model are presented in Table 5. The number of household members had a slight incremental effect on the likelihood of households using communal and farmland for food production (7% each). Households with higher dietary diversity were likely to use farmland for food procurement (10%). Distance to farms and greenspaces were significant predictors of use of greenspaces where food is procured. Increased distance to greenspaces predicted slightly lower household use of informal land (6%) and backyard gardens (7%), but greater use of farmland (7%), communal land (10%), and community gardens (15%) for food procurement. Lastly, increased distance to farms significantly reduced household use of farmland for food production (86%), and increased use of informal land slightly (2%).

**Table 5:** Predictors of household land access (binomial logistic models) and food security and dietary diversity (Poisson regression models). The 1-pχ^2^ value indicates Chi-squared goodness of fit for the model, and only significant predictors (*p* < 0.05) are reported for each model in this table. Full statistics of each regression model are presented in Appendix Tables 13–19

Land access (binomial model)	1-pχ^2^	Household attribute (predictor variable)	z	p-value	Log (estimate)
Informal Land	0.000513848	Food proximity index	2.023	0.05	1.015
		Distance to greenspaces	− 2.352	0.05	1.064
		Distance to farms	2.401	0.05	2.037
Communal Land	7.92E-11	Respondent age	2.491	0.05	1.070
		Food proximity index	− 3.274	0.01	1.023
		Distance to shops	− 2.777	0.01	1.039
		Distance to greenspaces	6.260	0.001	1.100
Communal Garden	1.42E-06	Distance to greenspaces	4.869	0.001	1.153
Backyard Garden	5.65E-06	Food security score	− 2.456	0.05	1.053
		Food proximity index	2.214	0.05	1.011
		Distance to shops	3.363	0.001	1.047
		Distance to greenspaces	− 4.553	0.001	1.068
Farm Land	1.39E-08	Household size	2.521	0.05	1.067
		Dietary diversity score	2.463	0.05	1.101
		Distance to shops	− 2.722	0.01	1.038
		Distance to greenspaces	4.940	0.001	1.069
		Distance to farms	− 2.256	0.05	1.857
Food security score (Poisson model)	3.55E-15	Wealth quintile	7.609	0.001	1.070
		Distance to greenspaces	− 3.125	0.01	1.004
		Distance to farms	3.444	0.001	1.071
Dietary diversity score (Poisson model)	0.00061421	Food proximity index	− 2.307	0.05	1.002
		Distance to shops	− 2.826	0.01	1.005
		Distance to farms	− 2.737	0.01	1.088

Poisson regression models are presented in Appendix Tables 18–19, and significant results from each model are reported in Table 5. Household distances to shops, greenspaces, and farms had small effects on household food security and dietary diversity. There was a 0.004 point reduction in food security score for every unit increment in distance in kilometres to greenspaces. For every unit increment in distance to farms, there was a 0.07 point increase in the food security score, and a 0.09 point reduction in the dietary diversity score. Every unit increment in distance to shops was linked to a 0.005 point reduction in dietary diversity score.

### Location: distances to shops, greenspaces, farms, and rural–urban gradient

In urban Kokstad, most respondent households in the west were located equally away from greenspaces, shops and farms. The distances to greenspaces and shops in the east were quite large, while farms were relatively close (Fig. 1E). Average distances in Kokstad were 1.7 km (± 0.5) to greenspaces, 1.5 km (± 0.4) to shops, and 1.1 km (± 0.4) to farms. In rural Nhlazuka, shops (27.2 km, ± 2.4) and greenspaces (23.2 km ± 2) were located very far away from most respondent households, but farms were relatively close (0.7 km ± 0.6, Fig. 1A), though not as dense as the other sites. In rural sites Swayimane (Fig. 1C) and Tugela Ferry (Fig. 1B), farms were located very close to all respondent households (0.02 km ± 0.04 and 0.5 km ± 0.3 respectively), while shops (3.3 km ± 1.3 and 3.8 km ± 1.4 respectively) were further away, and distances to greenspaces (11.5 km ± 1.8 and 24.8 km ± 0.5 respectively) were much greater. In peri-urban Umbumbulu, farms (0.1 km, ± 0.1), greenspace (1.6 km, ± 0.8), and shops (1.7 km, ± 0.9) were situated close to respondent households (Fig. 1D), similar to urban Kokstad.

In Kokstad, access to, and implied use of, farms, greenspaces, and shops resulted in high food security and proximity scores (Table 2). Notably, proximity to farms did not correspond to increased dietary diversity. Although the distance to greenspaces and shops was large for households in Nhlazuka (Fig. 1A), self-reported consumption strategies indicated that respondents preferred to procure food from shops, resulting in a low food proximity score (Table 2). Similar to urban Kokstad, proximity to farms in rural Nhlazuka did not correspond to greater dietary diversity. Households in Swayimane appeared to utilise their proximity to greenspaces and farms (Fig. 1C), resulting in significantly higher food proximity and dietary diversity scores (Table 2). Food proximity and dietary diversity scores were similar in rural Tugela Ferry and peri-urban Umbumbulu. However, food security scores were higher in the latter, ostensibly because of access to more shops in the wider vicinity, and mobility to approach these by road transport.

## Discussion

### Livelihoods and food and nutrition security

Our findings indicate that smallholder integrated crop-livestock farmers, particularly grain and poultry farmers, suffer from food and nutrition insecurity due to the nature of their livelihoods and location. The association between poultry and grain production likely represents the smallholder practice of growing grains, especially maize, to use as poultry feed (Sibanda et al. 2023). In South Africa, the poultry industry unintentionally marginalises smallholders by posing policy barriers to market participation (Queenan et al. 2022). Our findings on lower food and nutrition security among grain and poultry producers concur with the food security paradox, wherein subsistence farmers cannot attain nutrition security despite being food producers, due to market pressures favouring large-scale farming (Benton and Bailey 2019; Tomita et al. 2020, Giller 2020). Notably, household dietary diversity was significantly lower in poultry-producing households, and significantly higher in indigenous crop-growing households. Although the latter relationship could not be further investigated due to the small sub-sample size (*n* = 8), the inference is that crop diversity manifests in dietary diversity.

Integrated crop-livestock systems around much of the world have a history of being decoupled from lands and livelihoods in the interest of high-yielding, specialised, large-scale monocultures, including commodity crops (Garrett et al. 2020). Smallholders in the Global South, especially in South Asia and Africa, continue to integrate livestock with crops at farm scale and within the household economy; however, sub-Saharan smallholders are at significant risk of being marginalised due to low technological advances, greater market penetration by globalised food producers, and climate uncertainty (Giller et al. 2021). In recognition of this trend, agricultural extension in South Africa and other sub-Saharan countries must better equip smallholders to attain subsistence and nutrition security. This could be done by skilling smallholders to integrate indigenous breeds and crops, including fruits and vegetables, that are better adapted to local conditions (Mabhaudhi et al. 2019; Sardeshpande and Shackleton 2019; Sibanda et al. 2023); and enabling their participation in local and regional food economies by providing finance, infrastructure, and network support at scale (Thow et al. 2018; Garrett et al. 2020; Thinda et al. 2020). Food governance must consider smallholder empowerment when implementing agricultural policy for local food and nutrition security (Kushitor et al. 2022).

### Land access and food and nutrition security

Households with access to communal and informal land and gardens were more likely to be engaged in food production. However, those producing farmed ruminants were more food and nutrition secure than grain and poultry farmers. The implications of farming grain for poultry feed (Queenan et al. 2022; Sibanda et al. 2023), combined with the remoteness from non-farm food sources such as shops and gardens, likely exacerbate household nutrition insecurity. Communal livestock farming in the region has a legacy of spatial segregation and a related lack of alternative livelihoods (Khowa et al. 2023) and food supply infrastructure such as cold storage and shops, curtailing dietary options. Larger households were more likely to use communal and farmlands to produce and procure food. This may be a function of either greater availability of labour within the households to produce food, or of greater availability of funds from remittances from household members employed elsewhere to pay for labour and inputs (Ragie et al. 2020; Hlatshwayo et al. 2023).

Access to land is mediated by tenure, which influences how land is used for food production. Communal tenure lends itself to low-input livestock farming, but can deter more labour-intensive grain and fruit and vegetable farming due to risks related to accountability and spatial allocation (Bennett et al. 2013). Communal tenure may sometimes favour locally wealthy and powerful households who already possess assets (e.g. livestock, tractors), and capital to fund productive agriculture, undermining equitable benefit distribution (Olofsson 2021). Reduced certainty regarding land tenure and weather patterns, and increased dependence on remittances and purchased food have resulted in farm abandonment in parts of rural South Africa (Shackleton et al. 2019). Agrarian reform policies and extension services must recognise disparities between smallholders and commercial farmers (Gwiriri et al. 2019). Like our study, others have found that food gardens in communal and informal lands, and in homes, schools, and clinics, are significant sources of nutritious food from fresh fruits and vegetables in both rural and urban areas (Sardeshpande and Shackleton 2020; Hendriks et al. 2020; Gwedla et al. 2022). Government schemes for food and nutrition security, such as One Home One Garden and the National School Nutrition Scheme (Mensah and Karriem 2021), as well as greening initiatives such as Working for Ecosystems, should consider inclusion of indigenous and nutrient-dense species in publicly accessible gardens (Sardeshpande et al. 2021).

### Location and food and nutrition security

Food security increased slightly among houses closer to shops and further away from farms, but dietary diversity reduced along this gradient. Household dietary diversity increased with access and proximity to farms, and reduced with proximity to food provisioning shops (Table 5). This finding is also consistent with global trends in the overall homogenisation of diets and supply chains by industrialised and globalised food systems. Otterbach et al. (2021) found that proximity to food provisioning shops across South Africa can considerably increase the risk of malnutrition and related lifestyle diseases, irrespective of income, livelihood, and location. Complementing the food related-health implications of proximity to shops is our finding that non-farm greenspaces are important fresh food sources. When located at a distance from farms, significant portions of the sample used greenspaces such as backyard and community gardens and communal and informal lands for various types of food production (Table 5).

Overall, proximity to greenspaces, rather than farms, was linked to greater food access and utilisation (Fig. 1, Sect. 3.3). These findings link to the rising interest in food production in non-farm settings, such as communal and informal land, and food gardens (Sardeshpande et al. 2021; Russo and Cirella 2019). Fruit, vegetable, and indigenous crop farming can be feasible in small spaces, unlike grain and livestock farming, which require large tracts of land (Bennett and Lovell 2019, Agovino et al. 2018). Since these forms of food-producing livelihoods are also linked with better dietary diversity, there is a strong case for promoting fruit, vegetable, and indigenous crop production in smallholder farms and food gardens across the rural–urban continuum. Given that smaller households and smaller land parcels can support fruit and vegetable production with positive nutrition outcomes (Sect. 4.2, Giller et al. 2021), promoting such farming through agricultural extension is likely to improve livelihood prospects for rural farmers by diversifying their portfolio, both for household consumption and market sales. In urban areas, food gardens offering fruits and vegetables will likely counterbalance the impact of nutrient-poor calorie-dense foods (Otterbach et al. 2021; Downs et al. 2020).

## Policy implications

Our analyses reflect that food security was highest at the most urbanised site (Kokstad), and dietary diversity was greatest at the most agrarian site (Swayimane) (Table 2). This finding resonates with global trends in the literature, which point to a strong link between food-producing livelihoods and dietary diversity (Ricciardi et al. 2018; Abu Hatab et al. 2019). It also highlights that while industrialised agriculture and food supply chains (of which big farms and shops are a part) can improve food availability and access in urban areas, dietary diversity and nutrition security may be compromised by a singular focus on sufficiency. In contrast, rural areas may have greater potential for agricultural production and diverse food systems but may face significant challenges related to access to markets, resources, and infrastructure (Gashu et al. 2019). Findings from our study underscore the spatial complexities of the food security paradox, wherein, in some cases, households preferred to purchase food from stores despite their relative proximity to farms. This is ostensibly because farms may not grow food crops, may not sell produce to local buyers, or local buyers may not be able to access or afford food if grown on these farms.

Policy and extension that facilitates smallholder participation in markets and farm diversification is central to achieving equitable, healthy food systems and making farming a sustainable livelihood (Thinda et al. 2020; Kushitor et al. 2022). Even though policies such as the National Policy on Food and Nutrition Security for the Republic of South Africa (2014) addresses the challenges of food security in South Africa, and factors that affect food security, namely, availability, accessibility, utilisation and stability of food supplies, the role of extension services in policy implementation is not highlighted (DAFF 2014). Given the worsening food security in the country, it is important to include extension officers in the food security conversation, from policy to implementation. Furthermore, land reform and stewardship programmes should take into consideration the capital required to enable scaling of smallholder agriculture, including technical know-how, physical infrastructure, social and transport networks, and financial capital and instruments (Gashu et al. 2019, Sardeshpande et al. 2021). Food environments, defined as the cumulative environments including external drivers and intrinsic motivations that shape dietary choices, play a decisive role in nutrition outcomes (Downs et al. 2020). Environmental exposure to advertising and public health programming can influence dietary choices as much as exposure to farm landscapes and novel foods (Chen and Antonelli 2020). Creating healthy food environments in rural and urban areas will likely promote better food and nutrition outcomes. Food gardens can offer tenable alternatives to mainstream and commodity crops in rural areas (Hendriks et al. 2020), and to ultra-processed food outlets in urban areas (Rombach and Dean 2022). Knowledge is key to adopting nutritious diets (Chakona and Shackleton 2019), hence building awareness on nutrition-sensitive diets and dietary diversity is crucial. Public programming and experiential education through clinics, schools, and civil society organisations can aid in disseminating such information (Mensah and Karriem 2021).

### Limitations

While this research offers valuable insights into addressing food and nutritional security, some limitations may have impacted the generalizability of the findings. Firstly, the convenience sampling method used across the five sites may have introduced potential bias as participants were selected based on their willingness to participate. This did not allow for a systematic selection of the households. One of the outcomes of this sampling was that more male-headed, agrarian, and grant-recipient households participated in the study, which is not necessarily representative of all the communities. Secondly, the cross-sectional data collection may not have captured the long-term, seasonal fluctuations in food security trends. Thirdly, the use of HDDS as a proxy for household nutritional security is a coarse-scale indicator, and this variable could be better represented by the Food Consumption Score—Nutrition indicator (Leroy et al. 2015). Lastly, the food proximity score is a new concept that has not been tested with sufficiently large, diverse, or representative datasets for internal validity and endogeneity. Nevertheless, we posit that our research brings together insights using interdisciplinary techniques to frame food environments at different scales. To advance food and nutritional security research in the region, future studies should intentionally sample for better representativeness across socioeconomic categories, over time, and with more meaningful indicators. Longitudinal panel data such as the National Income Dynamics Survey (van der Berg et al. 2022) on livelihoods and health outcomes, does not delve into specificities of food and nutrition security and household location; however, future comparisons with these datasets may provide valuable insights for policy, planning, and implementation. This includes programming and interventions to improve farmer extension support, cooperative and market linkages, and nutrition and health education.

## Conclusion

Our study adds two dimensions to the understanding of food and nutrition security. One is that food-producing livelihoods better improve food and nutrition security when a diversity of food and income sources are accessible to the household. This includes food from non-farm greenspaces and public land, and income from off-farm livelihoods. The second is that overreliance on any single source of food is likely detrimental to food and nutrition security, irrespective of household wealth, livelihoods, or location along the rural–urban gradient. These dimensions are important to consider when viewed in light of the food security paradox and ongoing nutrition transitions.

Policy and programmes aimed at smallholder farmers and strengthening local food systems should enable on-farm diversification and financial, infrastructure, and training support to enhance local fresh food supply, in addition to the existing focus on food stocks and commercial value chains. Educating the public about fresh food through ongoing nutrition initiatives and living laboratories such as food gardens will bolster adoption of healthier diets, and create a market demand for fresh produce from local smallholders. Such localisation of food supply is likely to improve food system resilience in times of social-ecological shocks, improve food sovereignty, and contribute more effectively to local nutrition needs. Policy and planning across the rural–urban gradient should consider integrative approaches to land use, human settlements, and food and nutrition security outcomes.

## Data Availability

Data is provided within the manuscript or supplementary information files.

## Appendix

See Fig 2 and Tables

**Table 6:** List of variables and their distributions, means, and standard deviations

Variable	Type	Distribution	Mean	Std deviation
Respondent age (years)	Continuous	Non-normal	45	13.56
Respondent education level	Ordinal	Non-normal	2 Median = 2	0.92
Number of male adults per household	Count	Non-normal	2	1.68
Number of female adults per household	Count	Non-normal	3	1.88
Number of male children per household	Count	Non-normal	1	1.18
Number of female children per household	Count	Non-normal	1	1.30
Number of total members per household	Count	Non-normal	7	4.46
Number of earning members per household	Count	Non-normal	0.90	1.24
Household income category	Ordinal	Non-normal	4 Median = 4	1.20
Household food security score	Continuous	Non-normal	20.85	5.23
Household dietary diversity score	Continuous	Non-normal	9.40	3.34
Household food proximity index	Continuous	Non-normal	35.15	20.90
Distance from household to shops (kilometres)	Continuous	Non-normal	7.57	10.40
Distance from household to greenspaces (kilometres)	Continuous	Non-normal	12.78	10.11
Distance from household to farms (kilometres)	Continuous	Non-normal	0.47	0.52

^6^ and

**Table 7:** Distributions and percentages of survey respondent and household attributes

Level	Criteria	n	Percentage (*N* = 486)
Household	Heads		
	Female-headed	282	58
	Male-headed	111	23
	Joint headed	78	16
	Unknown	15	3
	Primary income		
	Support from family or neighbours	25	5
	Fulltime employment	85	18
	Parttime employment	102	21
	Pension	99	20
	Remittances	17	3
	Social government grants	135	28
	Unknown	18	4
	Other	5	1
	Agrarian livelihoods		
	Yes	291	60
	Grants		
	Yes	182	38
Respondent	Heads		
	Head respondent	371	76
	Non-head respondent	98	20
	Unknown	17	4
	Gender		
	Male	364	75
	Female	117	24
	Unknown	5	1
	Education		
	None	71	15
	Primary	133	28
	Secondary	181	37
	Tertiary	75	15
	Unknown	26	5

^7^

## Models

See Tables

**Table 8:** Predictors of agricultural production livelihood

Deviance residuals				
Min	1Q	Median	3Q	Max
− 2.0683	− 1.1633	0.7076	0.9911	1.6996

Coefficients				
	Estimate	Std. Error	z value	Pr( >|z|)
(Intercept)	− 1.358013	0.811057	− 1.674	0.0941
RespAge	0.017849	0.008233	2.168	0.0302 *
HHSize	0.044286	0.024237	1.827	0.0677
EduRank	0.097856	0.124673	0.785	0.4325
IncCat	− 0.015143	0.087813	− 0.172	0.8631
FoodSecScore	− 0.016993	0.020157	− 0.843	0.3992
NutScore	0.001416	0.030119	0.047	0.9625
FoodProxIndex	0.009121	0.004784	1.907	0.0566
DistShops	− 0.021777	0.012580	− 1.731	0.0835
DistGrSp	0.059158	0.013107	4.514	6.37e-06 ***
DistFarms	− 0.019788	0.195051	− 0.101	0.9192

Signif. codes: 0 ‘***’ 0.001 ‘**’ 0.01 ‘*’ 0.05 ‘.’ 0.1 ‘ ‘ 1

(Dispersion parameter for binomial family taken to be 1)

Null deviance: 654.65 on 485 degrees of freedom

Residual deviance: 612.45 on 475 degrees of freedom

AIC: 634.45

Number of Fisher Scoring iterations: 4

Model 1-pχ2 (Chi-squared goodness of fit): 6.918339E-06

^8^,

**Table 9:** Predictors of livestock production livelihood

Deviance residuals				
Min	1Q	Median	3Q	Max
− 0.9871	− 0.5493	− 0.4502	− 0.3629	2.4910

Coefficients				
	Estimate	Std. error	z value	Pr( >|z|)
(Intercept)	− 3.813944	1.196103	− 3.189	0.00143 **
RespAge	0.017843	0.011884	1.502	0.13322
HHSize	0.009763	0.030560	0.319	0.74936
EduRank	− 0.103745	0.179766	− 0.577	0.56386
IncCat	− 0.104187	0.127004	− 0.820	0.41202
FoodSecScore	0.023667	0.028419	0.833	0.40496
NutScore	0.003739	0.041200	0.091	0.92768
FoodProxIndex	0.010746	0.006620	1.623	0.10455
DistShops	0.041827	0.015531	2.693	0.00708 **
DistGrSp	0.015838	0.018894	0.838	0.40190
DistFarms	0.059091	0.281019	0.210	0.83345

Signif. codes: 0 ‘***’ 0.001 ‘**’ 0.01 ‘*’ 0.05 ‘.’ 0.1 ‘ ‘ 1

(Dispersion parameter for binomial family taken to be 1)

Null deviance: 374.88 on 485 degrees of freedom

Residual deviance: 353.27 on 475 degrees of freedom

AIC: 375.27

Number of Fisher Scoring iterations: 5

Model 1-pχ2 (Chi-squared goodness of fit): 0.01719307

^9^,

**Table 10:** Predictors of poultry production livelihood

Deviance residuals				
Min	1Q	Median	3Q	Max
− 1.7196	− 0.5489	− 0.4155	− 0.3152	2.5246

Coefficients				
	Estimate	Std. error	z value	Pr( >|z|)
(Intercept)	− 1.2470900	1.1347228	− 1.099	0.271757
RespAge	0.0259149	0.0118505	2.187	0.028756 *
HHSize	− 0.0166786	0.0315468	− 0.529	0.597018
EduRank	0.0439228	0.1807596	0.243	0.808013
IncCat	− 0.1376116	0.1253633	− 1.098	0.272334
FoodSecScore	− 0.0202140	0.0272201	− 0.743	0.457715
NutScore	− 0.1161649	0.0355297	− 3.270	0.001077 **
FoodProxIndex	0.0001419	0.0066504	0.021	0.982972
DistShops	0.0580755	0.0160009	3.630	0.000284 ***
DistGrSp	− 0.0039696	0.0194928	− 0.204	0.838633
DistFarms	− 0.6038788	0.3003655	− 2.010	0.044380 *

Signif. codes: 0 ‘***’ 0.001 ‘**’ 0.01 ‘*’ 0.05 ‘.’ 0.1 ‘ ‘ 1

(Dispersion parameter for binomial family taken to be 1)

Null deviance: 393.48 on 485 degrees of freedom

Residual deviance: 352.31 on 475 degrees of freedom

AIC: 374.31

Number of Fisher Scoring iterations: 5

Model 1-pχ2 (Chi-squared goodness of fit): 1.053977E-05

^10^,

**Table 11:** Predictors of grain production livelihood

Deviance residuals				
Min	1Q	Median	3Q	Max
− 1.6519	− 0.8923	− 0.5392	1.0421	2.2542

Coefficients				
	Estimate	Std. error	z value	Pr( >|z|)
(Intercept)	− 3.414283	0.901078	− 3.789	0.000151 ***
RespAge	0.047155	0.009528	4.949	7.45e-07 ***
HHSize	0.031733	0.024234	1.309	0.190375
EduRank	0.470462	0.138045	3.408	0.000654 ***
IncCat	0.070202	0.100288	0.700	0.483927
FoodSecScore	− 0.028779	0.021798	− 1.320	0.186751
NutScore	− 0.013076	0.031285	− 0.418	0.675974
FoodProxIndex	0.001096	0.005234	0.209	0.834188
DistShops	− 0.045850	0.013723	− 3.341	0.000834 ***
DistGrSp	0.054751	0.013411	4.083	4.45e-05 ***
DistFarms	− 1.021274	0.255514	− .997	6.42e-05 ***

Signif. codes: 0 ‘***’ 0.001 ‘**’ 0.01 ‘*’ 0.05 ‘.’ 0.1 ‘ ‘ 1

(Dispersion parameter for binomial family taken to be 1)

Null deviance: 624.09 on 485 degrees of freedom

Residual deviance: 537.32 on 475 degrees of freedom

AIC: 559.32

Number of Fisher Scoring iterations: 4

Model 1-pχ2 (Chi-squared goodness of fit): 2.342571E-14

^11^,

**Table 12:** Predictors of fruit and vegetable production livelihood

Deviance residuals				
Min	1Q	Median	3Q	Max
− 1.2503	− 0.7359	− 0.6179	− 0.4455	2.3487

Coefficients				
	Estimate	Std. error	z value	Pr( >|z|)
(Intercept)	0.469479	0.910816	0.515	0.6062
RespAge	− 0.005303	0.009433	− 0.562	0.5740
HHSize	− 0.067612	0.030285	− 2.232	0.0256 *
EduRank	− 0.295639	0.142967	− 2.068	0.0387 *
IncCat	− 0.060040	0.100478	− 0.598	0.5501
FoodSecScore	− 0.005593	0.022294	− 0.251	0.8019
NutScore	− 0.056558	0.032318	− 1.750	0.0801
FoodProxIndex	0.004885	0.005396	0.905	0.3653
DistShops	− 0.024072	0.014386	− 1.673	0.0943
DistGrSp	0.015620	0.013895	1.124	0.2610
DistFarms	0.236430	0.225758	1.047	0.2950

Signif. codes: 0 ‘***’ 0.001 ‘**’ 0.01 ‘*’ 0.05 ‘.’ 0.1 ‘ ‘ 1

(Dispersion parameter for binomial family taken to be 1)

Null deviance: 514.87 on 485 degrees of freedom

Residual deviance: 494.63 on 475 degrees of freedom

AIC: 516.63

Number of Fisher Scoring iterations: 4

Model 1-pχ2 (Chi-squared goodness of fit): 0.02702448

^12^,

**Table 13:** Predictors of informal land use

Deviance residuals				
Min	1Q	Median	3Q	Max
− 1.0178	− 0.4596	− 0.3429	− 0.2465	2.7021

Coefficients				
	Estimate	Std. error	z value	Pr( >|z|)
(Intercept)	− 3.430342	1.093025	− 3.138	0.0017 **
HHSize	0.026989	0.036622	0.737	0.4612
IncCat	− 0.099534	0.146630	− 0.679	0.4973
FoodSecScore	0.051509	0.037929	1.358	0.1745
NutScore	− 0.026350	0.048140	− 0.547	0.5841
FoodProxIndex	0.015035	0.007433	2.023	0.0431 *
DistShops	0.016353	0.026397	0.620	0.5356
DistGrSp	− 0.061913	0.026322	− 2.352	0.0187 *
DistFarms	0.711415	0.296281	2.401	0.0163 *

Signif. codes: 0 ‘***’ 0.001 ‘**’ 0.01 ‘*’ 0.05 ‘.’ 0.1 ‘ ‘ 1

(Dispersion parameter for binomial family taken to be 1)

Null deviance: 295.27 on 485 degrees of freedom

Residual deviance: 267.47 on 477 degrees of freedom

AIC: 285.47

Number of Fisher Scoring iterations: 6

Model 1-pχ2 (Chi-squared goodness of fit): 0.0005138475

^13^,

**Table 14:** Predictors of communal land use

Deviance residuals				
Min	1Q	Median	3Q	Max
− 1.4036	− 0.6461	− 0.4599	− 0.2963	2.7374

Coefficients				
	Estimate	Std. error	z value	Pr( >|z|)
(Intercept)	− 3.108682	0.824074	− 3.772	0.000162 ***
HHSize	0.067353	0.027041	2.491	0.012746 *
IncCat	− 0.054185	0.112417	− 0.482	0.629804
FoodSecScore	0.025523	0.025120	1.016	0.309613
NutScore	0.058371	0.040600	1.438	0.150525
FoodProxIndex	− 0.022911	0.006997	− 3.274	0.001060 **
DistShops	− 0.038236	0.014596	− 2.777	0.005488 **
DistGrSp	0.091379	0.026322	6.260	3.84e-10 ***
DistFarms	0.017082	0.269065	0.063	0.949380

Signif. codes: 0 ‘***’ 0.001 ‘**’ 0.01 ‘*’ 0.05 ‘.’ 0.1 ‘ ‘ 1

(Dispersion parameter for binomial family taken to be 1)

Null deviance: 485.84 on 485 degrees of freedom

Residual deviance: 421.92 on 477 degrees of freedom

AIC: 439.92

Number of Fisher Scoring iterations: 5

Model 1-pχ2 (Chi-squared goodness of fit): 7.917311e-11

^14^,

**Table 15:** Predictors of communal garden use

Deviance residuals				
Min	1Q	Median	3Q	Max
− 1.1156	− 0.4422	− 0.2130	− 0.1353	3.0354

Coefficients				
	Estimate	Std. error	z value	Pr( >|z|)
(Intercept)	− 4.639728	1.310536	− 3.540	0.0004 ***
HHSize	− 0.060547	0.055662	− 1.088	0.2767
IncCat	− 0.289529	0.164915	− 1.756	0.0792
FoodSecScore	0.048954	0.037013	1.323	0.1860
NutScore	0.042072	0.059977	0.701	0.4830
FoodProxIndex	− 0.004521	0.010456	− 0.432	0.6655
DistShops	− 0.024437	0.018656	− 1.310	0.1902
DistGrSp	0.142478	0.029261	4.869	1.12e-06 ***
DistFarms	0.019055	0.434493	0.044	0.9650

Signif. codes: 0 ‘***’ 0.001 ‘**’ 0.01 ‘*’ 0.05 ‘.’ 0.1 ‘ ‘ 1

(Dispersion parameter for binomial family taken to be 1)

Null deviance: 246.43 on 485 degrees of freedom

Residual deviance: 204.55 on 477 degrees of freedom

AIC: 222.55

Number of Fisher Scoring iterations: 7

Model 1-pχ2 (Chi-squared goodness of fit): 1.424485e-06

^15^,

**Table 16:** Predictors of backyard garden use

Deviance residuals				
Min	1Q	Median	3Q	Max
− 1.4435	− 0.9034	− 0.6562	1.2484	2.1114

Coefficients				
	Estimate	Std. error	z value	Pr( >|z|)
(Intercept)	0.21535	0.63643	0.338	0.735081
HHSize	0.01147	0.02292	0.500	0.616841
IncCat	0.15572	0.09573	1.627	0.103801
FoodSecScore	− 0.05133	0.02090	− 2.456	0.014038 *
NutScore	− 0.05473	0.03046	− 1.797	0.072366
FoodProxIndex	0.01078	0.00487	2.214	0.026823 *
DistShops	0.04603	0.01369	3.363	0.000771 ***
DistGrSp	− 0.06579	0.01445	− 4.553	5.29e-06 ***
DistFarms	− 0.17150	0.20490	− 0.837	0.402612

Signif. codes: 0 ‘***’ 0.001 ‘**’ 0.01 ‘*’ 0.05 ‘.’ 0.1 ‘ ‘ 1

(Dispersion parameter for binomial family taken to be 1)

Null deviance: 602.31 on 485 degrees of freedom

Residual deviance: 563.63 on 477 degrees of freedom

AIC: 581.63

Number of Fisher Scoring iterations: 4

Model 1-pχ2 (Chi-squared goodness of fit): 5.645312e-06

^16^,

**Table 17:** Predictors of farm land use

Deviance residuals				
Min	1Q	Median	3Q	Max
− 1.7906	− 0.7659	− 0.5351	− 0.2928	2.3960

Coefficients				
	Estimate	Std. error	z value	Pr( >|z|)
(Intercept)	− 3.215127	0.771488	− 4.167	3.08e-05 ***
HHSize	0.064704	0.025670	2.521	0.01172 *
IncCat	0.034999	0.107177	0.327	0.74401
FoodSecScore	− 0.002867	0.023282	− 0.123	0.90198
NutScore	0.096143	0.039032	2.463	0.01377 *
FoodProxIndex	0.004114	0.005638	0.730	0.46564
DistShops	− 0.037619	0.013822	− 2.722	0.00649 **
DistGrSp	0.066771	0.013517	4.940	7.82e-07 ***
DistFarms	− 0.618793	0.274290	− 2.256	0.02407 *

Signif. codes: 0 ‘***’ 0.001 ‘**’ 0.01 ‘*’ 0.05 ‘.’ 0.1 ‘ ‘ 1

(Dispersion parameter for binomial family taken to be 1)

Null deviance: 534.17 on 485 degrees of freedom

Residual deviance: 481.74 on 477 degrees of freedom

AIC: 499.74

Number of Fisher Scoring iterations: 4

Model 1-pχ2 (Chi-squared goodness of fit): 1.389181e-08

^17^,

**Table 18:** Predictors of household food security

Deviance residuals				
Min	1Q	Median	3Q	Max
− 6.1012	− 0.5680	0.1062	0.7685	2.0815

Coefficients				
	Estimate	Std. error	z value	Pr( >|z|)
(Intercept)	2.8135077	0.0758080	37.114	< 2e-16 ***
RespAge	− 0.0013533	0.0008336	− 1.624	0.104478
HHSize	− 0.0028400	0.0023321	− 1.218	0.223297
EduRank	0.0101931	0.0126776	0.804	0.421381
IncCat	0.0677692	0.0089065	7.609	2.76e-14 ***
NutScore	0.0050664	0.0031001	1.634	0.102208
FoodProxIndex	− 0.0002328	0.0004900	− 0.475	0.634691
DistShops	0.0007273	0.0012716	0.572	0.567335
DistGrSp	− 0.0039952	0.0012784	− 3.125	0.001777 **
DistFarms	0.0687795	0.0199714	3.444	0.000573 ***

Signif. codes: 0 ‘***’ 0.001 ‘**’ 0.01 ‘*’ 0.05 ‘.’ 0.1 ‘ ‘ 1

(Dispersion parameter for binomial family taken to be 1)

Null deviance: 769.31 on 485 degrees of freedom

Residual deviance: 680.97 on 476 degrees of freedom

AIC: 3046.5

Number of Fisher Scoring iterations: 4

Model 1-pχ2 (Chi-squared goodness of fit): 3.552714e-15

^18^,

**Table 19:** Predictors of household dietary diversity

Deviance residuals				
Min	1Q	Median	3Q	Max
− 4.5761	− 0.3946	0.3799	0.6677	1.4875

Coefficients				
	Estimate	Std. error	z value	Pr( >|z|)
(Intercept)	2.4134375	0.1122338	21.504	< 2e-16 ***
RespAge	− 0.0015637	0.0012482	− 1.253	0.21030
HHSize	− 0.0043985	0.0035527	− 1.238	0.21570
EduRank	0.0015202	0.0189388	0.080	0.93603
IncCat	− 0.0142208	0.0135356	− 1.051	0.29344
FoodSecScore	0.0050238	0.0030815	1.630	0.10304
FoodProxIndex	− 0.0016838	0.0007298	− 2.307	0.02103 *
DistShops	− 0.0053799	0.0019039	− 2.826	0.00472 **
DistGrSp	0.0011408	0.0018915	0.603	0.54645
DistFarms	− 0.0840996	0.0307214	− 2.737	0.00619 **

Signif. codes: 0 ‘***’ 0.001 ‘**’ 0.01 ‘*’ 0.05 ‘.’ 0.1 ‘ ‘ 1

(Dispersion parameter for binomial family taken to be 1)

Null deviance: 893.72 on 485 degrees of freedom

Residual deviance: 864.58 on 476 degrees of freedom

AIC: 2761

Number of Fisher Scoring iterations: 4

Model 1-pχ2 (Chi-squared goodness of fit): 0.0006142113

^19^ and

**Table 20:** Spearman-Rank correlations to determine variable collinearity

Variable 1	Variable 2	rho	p	S
Food Security	FoodProxInd	− 0.03620436	0.4258	19,824,449
Food Security	NutScore	0.1384757	**0.002216**	16,482,507
Food Security	DistShops	− 0.123952	**0.006217**	21,503,220
Food Security	DistGrSp	− 0.1093076	**0.01592**	21,223,045
Food Security	DistFarms	0.11327	**0.01246**	16,964,736
Food Proximity Index	NutScore	− 0.04748935	**0.2961**	20,040,352
Food Proximity Index	DistShops	− 0.06264306	**0.168**	20,330,269
Food Proximity Index	DistGrSp	− 0.06866168	**0.1306**	20,445,416
Food Proximity Index	DistFarms	− 0.1328581	**0.003342**	21,673,610
Nutrition Score	DistShops	− 0.07289008	**0.1085**	20,526,313
Nutrition Score	DistGrSp	− 0.01441491	**0.7513**	19,407,578
Nutrition Score	DistFarms	− 0.1791699	**7.13E-05**	22,559,637

The bold values indicate a p-value of < 0.05, which renders the relationship statistically significant

20
